# Navigating the systemic labyrinth: the dual-track breakthrough of student-athletes under China's “Integration of Sports and Education”

**DOI:** 10.3389/fpsyg.2026.1882691

**Published:** 2026-09-29

**Authors:** Yinru Chen, Lin Li

**Affiliations:** 1 School of Education, Beijing Sport University, Beijing, China; 2 Capital University of Physical Education and Sports, Beijing, China

**Keywords:** dual-career, educational opportunity, Integration of Sports and Education, psychosocial adaptation, student-athletes

## Abstract

**Introduction:**

Although dual career (DC) development has received growing international attention, the psychosocial experiences of high-level student-athletes (HLSAs) within China's evolving Integration of Sports and Education (ISE) context remain underexplored.

**Methods:**

This study adopted an interpretivist qualitative design and used Reflexive Thematic Analysis to examine retrospective developmental accounts from 19 Chinese HLSAs admitted to university through the high-level sports team pathway.

**Results:**

Athletic achievement expanded access to higher education but did not automatically secure stable competence, legitimacy, or belonging across academic and athletic settings. Effort often remained within HLSAs' control even when its returns became uncertain because of injury, bodily limits, competitive conditions, and fluctuating performance, requiring athletes to recalibrate causal expectations and perceptions of what could still be changed. Institutional support, recognition, and opportunities also varied with athletic standing, making the quality of integration psychologically consequential beyond the formal coexistence of sport and education.

**Discussion:**

These findings conceptualize DC as a process of conditional inclusion. Chinese HLSAs navigate a systemically structured but psychologically experienced labyrinth, in which breakthrough occurs when athletic achievement is transformed from an entry credential and performance identity into a broader basis for competence, belonging, self-redefinition, and future possibility. Effective ISE therefore requires developmental conditions that allow sporting achievement to open educational opportunities without defining the limits of student-athletes' subsequent academic, personal, and career development.

## The global context: navigating the dual-career challenge

In high-performance sport, a dual career (DC) refers to the concurrent pursuit of athletic and educational development and has become an established focus within athlete career research (Stambulova and Wylleman, 2019; Stambulova et al., 2024). Sustaining high-level commitments to both sport and education can generate time pressure, fatigue, role conflict, and identity tensions, while inadequate support may intensify the strain associated with these competing demands (Condello et al., 2019; Stambulova et al., 2024).

In Europe, this has included the development of DC-friendly guidelines, minimum quality requirements for support services, flexible academic arrangements, and coordinated institutional support intended to enable athletes to remain engaged in both sport and education (Abelkalns et al., 2021; Condello et al., 2019; European Commission, 2013, 2016; Ryba et al., 2017). In the United States, collegiate sport has developed within the distinct organizational and regulatory structure of the NCAA, which institutionalizes the coexistence of higher education and competitive sport while also generating its own tensions surrounding academic and athletic priorities (Comeaux and Harrison, 2011; Southall and Staurowsky, 2013).

Such arrangements are embedded in particular educational systems, sport structures, and cultural expectations. Developmental trajectories and identity negotiations cannot be assumed to operate identically across national contexts (Stambulova et al., 2021). Comparatively less is known about how DC has been experienced within China's state-directed sport and education system across the broader institutional transition surrounding the introduction and implementation of the “Integration of Sports and Education” (ISE). Examining this context provides an opportunity to understand how a distinct institutional configuration shapes student-athletes' identity negotiation, responses to structural constraints, and the ways in which athletic experience is converted into broader developmental resources.

## The Chinese context: institutional paradigms of student-athlete cultivation and the cultural imperative of Wen-Wu Shuangquan

To understand the developmental trajectories of Chinese student-athletes, their experiences need to be situated within the country's distinctive institutional and cultural context. Historically, elite athletic preparation under the state-sponsored Juguo Tizhi and mainstream education largely developed through separate institutional systems (Zheng et al., 2018). In response to longstanding tensions between sport training and mainstream education, China introduced the “Integration of Sports and Education” (ISE) policy framework in 2020 (Sun et al., 2025). The national Opinions on Deepening the Integration of Sports and Education to Promote the Healthy Development of Youth, issued on August 31, 2020 and publicly released the following month, called for closer coordination between academic learning, physical development, and youth development (General Administration of Sport of China, and Ministry of Education of the People's Republic of China, 2020). A further reform of the high-level sports team admission system was announced in 2021, with major measures taking effect from 2024, including the national Gaokao for all high-level sports team applicants, nationally standardized sport testing, higher academic thresholds, and revised athlete eligibility requirements (Ministry of Education of the People's Republic of China, and General Administration of Sport of China, 2021). Participants in the present study entered university between 2016 and 2025; their retrospective accounts span different points within this broader institutional transition rather than a single ISE policy regime. ISE operates as a nationwide policy framework through which education and sport authorities coordinate resources, training, competition, and educational provision according to their respective institutional responsibilities. At the university level, one important component of this framework is the development of high-level sports teams and academic arrangements that enable student-athletes to pursue competitive sport without lowering educational standards.

Within this broader policy framework, the high-level sports team admission pathway provides a particularly important institutional route through which athletic achievement and higher education are connected. Regular university admission is based primarily on candidates' performance in the National College Entrance Examination (Gaokao) and does not involve recruitment through a university high-level sports team. The high-level sports team pathway—the route relevant to the HLSAs examined in this study—combines academic admission requirements with sport-specific eligibility and competitive performance criteria. A separate sports-specialist admission route (Tiyu Danzhao) uses its own cultural examination and sport-specific assessment and is primarily linked to designated sport-related majors; athletes admitted through this route were not included in the present study. The 2021 Guiding Opinions on Further Improving and Regulating the Examination and Admission of High-Level Sports Teams in Higher Education further formalized the dual academic-athletic requirements of the high-level sports team pathway and extended regulation from admission to post-entry training and academic management (Ministry of Education of the People's Republic of China, and General Administration of Sport of China, 2021). The reform also requires universities operating high-level sports teams to maintain academic standards while providing mechanisms such as credit-based arrangements, extended study periods, individualized instruction, and supplementary classes to help athletes manage concurrent training and educational obligations. Admission through this pathway requires both sport-specific eligibility and academic thresholds within China's university admissions system.

These institutional expectations intersect with a longstanding socio-cultural ideal often expressed as Wen-Wu Shuangquan. This ideal draws on the enduring wen–wu dyad in Chinese thought, which juxtaposes and connects civil or literary cultivation (wen) with martial qualities (wu) (Benesch, 2011). At the same time, contemporary Chinese schooling has been strongly shaped by an exam-oriented educational culture in which academic progression and entrance-examination performance carry substantial social significance (Dello-Iacovo, 2009). This intersection provides the specific context in which their DC identities, adaptations, and developmental experiences examined in this study take shape.

### Research gaps and the present study

Despite the burgeoning interest in DC pathways globally, several critical gaps remain in the extant literature, particularly concerning the Chinese context. Chinese elite sport has historically developed within the state-led Juguo Tizhi, which has strongly shaped the institutional organization of high-performance sport (Zheng et al., 2018). HLSAs in the university sector represent a distinct group whose experiences remain under-researched. HLSAs in the university sector represent a distinct group whose experiences remain under-researched. These athletes occupy an institutional position in which academic admission requirements and high-level athletic qualifications intersect. Chinese higher education does not prescribe a single national GPA threshold for all university students; assessment, credit progression, and graduation requirements are determined by individual institutions (Ministry of Education of the People's Republic of China, 2017). For HLSAs, national policy requires that academic standards should, in principle, remain consistent with those applied to other students at the same institution (Ministry of Education of the People's Republic of China, and General Administration of Sport of China, 2021).

International DC research has generated a substantial body of evidence on student-athletes' academic and athletic motivation, identity, career transitions, institutional support, and lived experiences. These issues have been examined through quantitative approaches (Gaston-Gayles, 2005; Lupo et al., 2015; Quinaud et al., 2020; Lee et al., 2022), qualitative inquiry (Harrison and Lawrence, 2004; Capranica et al., 2022), and longitudinal mixed-methods research (Thompson et al., 2024). Recent reviews have further synthesized student-athletes' perspectives on DC policies, provisions, and challenges (De Maio et al., 2025). The remaining research gap is limited understanding of how these processes unfold among Chinese HLSAs within the specific institutional context of ISE. In particular, little is known about how HLSAs interpret and navigate the intertwined academic, athletic, psychosocial, and institutional demands they encounter across their developmental trajectories. An interpretive qualitative approach allows closer examination of how these experiences are understood within the underexplored Chinese context.

A further gap concerns the relationship between policy structures and lived DC experiences. Capranica and Guidotti (2016) conceptualize DC as a multilevel phenomenon involving the individual athlete (micro), interpersonal relationships (meso), sport and education environments (macro), and organizational and governmental policy structures. Although ISE establishes a national policy framework for coordinating sport and education, less is known about how HLSAs experience and negotiate these expectations within their everyday academic, athletic, and institutional environments. The present study examines how individual experiences of identity negotiation, adaptation, and development intersect with the interpersonal and institutional conditions through which ISE is encountered in practice.

### Psychosocial processes within institutional contexts

This study frames Chinese HLSAs' DC experiences as psychosocial processes embedded within institutional contexts. The analysis focuses on identity negotiation, interpretations of setbacks and bodily limits, responses to stigma, and the meanings attached to athletic and educational experiences. These processes unfold through athletes' experiences of relationships, organizational arrangements, and institutional expectations that shape access to resources, recognition, and opportunities.

### Research questions

The study focuses on how HLSAs negotiate identity and respond to psychosocial challenges within changing institutional conditions: how do HLSAs in Chinese universities perceive and negotiate their dual identities (student vs. athlete) throughout their developmental trajectories? What psychosocial challenges do HLSAs experience within these institutional conditions, and how do they interpret and respond to them across their developmental histories?

## Methodology

### Research paradigm and reflexive positionality

This study was approved by the relevant institutional ethics committee (details blinded for peer review). All participants provided written informed consent before participation in the study. This study adopted an interpretivist paradigm and Reflexive Thematic Analysis (RTA; Braun and Clarke, 2022), treating meaning as situated and co-constructed through the researcher's interpretive engagement with the data.

In RTA, reflexivity requires explicit attention to how the researcher's theoretical commitments, experiences, and interpretive choices shape the analytical process (Dodgson, 2019; Braun and Clarke, 2021). Reflexivity focused on how the researcher's theoretical commitments, experiences, and interpretive choices shaped the analysis (Dodgson, 2019; Braun and Clarke, 2021).

The primary researcher occupied an “insider” position as a former student-athlete who had personally experienced the dual-track system, including participation in the Gaokao admission process for high-level sports teams. The researcher's subsequent academic trajectory from undergraduate to doctoral study in sports science also provided sustained familiarity with the institutional context of ISE and the experiences of HLSAs. Throughout the RTA process, this experiential background was treated as an analytical resource while remaining subject to ongoing reflexive scrutiny. Interpretations were developed through engagement with participants' narratives and the broader institutional context of ISE and holistic human development.

The first author's insider familiarity with the dual-track system also sensitized the analysis to issues such as academic–athletic tension, institutional support, identity negotiation, and social perceptions. Likewise, the wording and sequencing of interview questions shaped the topics participants were invited to reflect upon. These influences were treated as part of the conditions through which the data and themes were constructed. Analytical memos were used to examine occasions where prior experience or theoretical concepts appeared to be directing interpretation beyond what the data supported, and candidate interpretations were subsequently narrowed, revised, or rejected where necessary (Supplementary Appendix B).

### Participants and context

Participants were recruited through criterion-based purposive and maximum variation sampling. Maximum variation sampling was used to capture a broad range of experiences across gender, sport, enrollment year, and current developmental position, and recruitment channels included campus networks, social media recruitment posts, and peer referrals. A total of 25 eligible individuals were approached. One declined participation, and 24 completed an interview. Five completed interviews were excluded during transcript review and data familiarization because they did not provide sufficient contextualized experiential material to address the research questions. Three contained persistently brief or evasive responses; one contained substantial gaps in the developmental account that could not be clarified because the participant declined a follow-up interview; and one consisted largely of generalized evaluative and affective statements without sufficient descriptions of specific academic or athletic experiences. These exclusions were made before formal theme development and were based on data adequacy rather than the substantive position or evaluative stance expressed by participants, resulting in a final analytic sample of 19 HLSAs. No participant withdrew after providing consent. The primary researcher had no pre-existing personal, professional, or supervisory relationship with the participants; contact was established specifically for the present study. Participants received a modest cash honorarium after the interview as compensation for their time.

All participants met the athlete technical-grade requirements applicable to their year of admission; the minimum qualification represented in the sample was National Second-Class Athlete. Athletes admitted through the separate Tiyu Danzhao pathway or those who remained exclusively within the full-time state-sponsored professional sport system during senior secondary education were excluded. The sample was limited to HLSAs who had successfully entered higher education through this pathway.

The final sample comprised 19 HLSAs: 10 males (52.6%) and 9 females (47.4%). Thirteen participants (68.4%) competed in individual sports, including track and field, swimming, tennis, fencing, badminton, and table tennis, and six (31.6%) competed in team sports, including volleyball, soccer, and basketball. Undergraduate majors were grouped into Sports/Physical Activity (*n* = 8, 42.1%), Business/Economics (*n* = 5, 26.3%), Communication (*n* = 3, 15.8%), and Humanities/Social Sciences (*n* = 3, 15.8%).

Participants entered university between 2016 and 2025, spanning a broader period of institutional transition in China's sport–education policy and high-level sports team admission system. Their experiences therefore reflect different policy contexts across this period rather than a single ISE regime. At the time of interview, seven participants (36.8%) were undergraduates, six (31.6%) were graduate students, four (21.1%) were university faculty members, one (5.3%) was a coach, and one (5.3%) was a professional athlete. All participants were assigned alphanumeric pseudonyms to protect confidentiality. Participant characteristics are summarized in Table 1.

**Table 1:** Participant characteristics of the interviewed HLSAs (*N* = 19).

Variable	Category	*N*
Gender	Male/female	10/9
Sport type	Individual/team	13/6
Current status	Undergraduate/graduate student/employed	7/6/6
Academic field	Sports/physical activity/business/economics/communication/humanities/social sciences	8/5/3/3
Interview duration	60/90/120 min	14/4/1

Participants enrolled between 2016 and 2025, spanning a broader period of institutional transition in China's sport-education policy.

### Data collection and instrument development

The semi-structured interview guide was developed iteratively from the literature and study aims. An initial version organized questions around participant background, developmental experiences, and meaning/value, with attention to key people, events, and settings. Following discussions with experts, teachers, and peers, the guide was revised and then tested in two pilot interviews. The pilots prompted greater attention to non-linear trajectories, participant-defined developmental stages, recurring experiences, and institutional and policy contexts. The final guide comprised 17 core questions with follow-up prompts and is provided in Supplementary Appendix A. Some prompts explicitly invited participants to discuss experiences of unfair treatment, misunderstanding, or social perceptions, while others elicited supportive experiences, developmental turning points, identity, perceived value, and open-ended reflections. Participants received the interview guide and informed-consent materials approximately 12 h before the interview. Because advance access could shape salience and preparation, interview accounts were treated as situated and co-constructed.

### Data analysis: a reflexive thematic approach

The Chinese-language dataset was analyzed manually in Microsoft Word and Excel using Braun and Clarke's (2022) six-phase RTA framework. Initial coding was predominantly inductive and semantic. Theme development became increasingly abductive and latent, with selected theoretical concepts introduced iteratively as interpretive resources rather than a priori coding categories.

The first author conducted all interviews, transcript checking, initial coding, analytical memoing, and primary theme development. The second author contributed during theme refinement through critical peer dialogue focused on the conceptual boundaries, wording, and evidential grounding of the 14 sub-themes. Supplementary Appendix B provides illustrative examples of the progression from data extracts to initial codes, candidate sub-themes, and final themes.

All coding, memoing, and theme development were conducted in Chinese using the original interview transcripts. Quotations selected for presentation were subsequently translated into English by the first author and checked against the Chinese originals by the second author, a university English instructor with professional language training. Translation prioritized close correspondence with participants' wording and meaning, with minor adjustments only for English readability. Metaphors and culturally specific expressions were retained wherever their meaning could be conveyed in English.

### Rigor and trustworthiness

Braun and Clarke's 15-point checklist was used as a reflexive prompt for reviewing the coherence and transparency of the analysis. Analytical memos were used to interrogate emerging interpretations and the influence of the first author's prior experience and theoretical commitments. For example, an early interpretation of one participant's experiences through the concepts of learned helplessness and “hollow-heart” distress was reconsidered after returning to the transcript and comparing the case with the wider dataset. These concepts were judged to be more clinically and theoretically loaded than the evidence warranted and were not retained in the final thematic structure (Supplementary Appendix B).

Two participants were invited to reflect on whether the developing interpretation resonated with their experiences and whether they perceived important misrepresentations. Their responses were treated as additional reflexive input. Critical peer dialogue with the second author further examined the conceptual boundaries, wording, and evidential fit of the 14 sub-themes. Seven working labels were subsequently refined where their wording was overly absolute, conceptually conflated, or more theoretically expansive than the data supported.

Thick description and illustrative extracts were used to make the relationship between data and interpretation visible and to enable readers to assess the relevance and transferability of the findings.

### Analytical overview: thematic integration

The RTA of the 19 HLSAs' narratives generated 14 sub-themes, which were conceptually integrated into four overarching themes (Table 2). These themes capture recurrent patterns across participants' retrospective accounts and provide the analytical structure for the findings that follow. Some meanings appeared at different points in participants' developmental histories. As mobility could operate as an anticipated motivation for university entry and later as a perceived educational or occupational outcome.

**Table 2:** The thematic integration matrix: mapping 14 sub-themes to 4 overarching themes.

Overarching theme	Integrated sub-themes	Core organizing concept and analytical function
Theme 1. The paradox of sports capital: instrumental rationality and dual-track identity negotiation	ST1. Growth trajectories and shifting academic–athletic priorities	Educational access and identity negotiation: captures how athletic achievement operates as a pragmatic route to educational opportunity while also acquiring changing personal and identity meanings. Participants negotiate competence, legitimacy, and belonging across academic and athletic settings as their priorities and relationships with sport change over time.
	ST3. Contextual identity negotiation and psychological transition	
	ST8a. Anticipated educational mobility as entry motivation	
	ST10. Instrumental use of sport for educational access	
Theme 2. Navigating the systemic labyrinth: uneven support, regulation, and performance-contingent recognition	ST2. Uneven support and resource allocation	Institutional conditions and psychosocial experience: examines how support, regulation, resources, and recognition vary across institutional settings and with athletic standing. These conditions shape athletes' experiences of competence, belonging, adjustment, and access to developmental opportunities.
	ST7. Academic-athletic conflict and institutional coordination	
	ST11. Regulated training environments and re-adaptation	
	ST12. Performance-contingent recognition, opportunity, and team status	
Theme 3. Navigating uncertainty, informal practices, and multidimensional challenges	ST4. Challenges, conflicts, and adaptation strategies	Control, uncertainty, and adaptation: examines how participants interpret uncertain returns to effort, bodily and competitive limits, perceived unfairness, injury, and academic difficulty. Adaptation involves differing judgments about controllability, causal expectations, and what can still be changed.
	ST13. Informal practices and perceived unfairness	
	ST14. Talent-effort tensions and perceived competitive limits	
Theme 4. Beyond the field: perceived educational and occupational mobility, identity legitimacy, and developmental meanings	ST5. Sports and human development	Mobility, legitimacy, and broader development: brings together accounts of educational and occupational opportunity, contested student-athlete legitimacy, policy interpretation, identity reconstruction, and meanings of sport that extend beyond immediate athletic performance.
	ST6. Perceptions of policy change and pathway legitimacy	
	ST8b. Perceived educational and occupational mobility	
	ST9. Student-athlete stereotypes and legitimacy claims	

ST8 appears in two analytical positions because participants described mobility both prospectively, as a motivation for university entry (ST8a), and retrospectively, as a perceived educational or occupational outcome (ST8b).

### Theme 1. The paradox of sports capital: instrumental rationality and dual-track identity negotiation

Sport could function as a pragmatic route to educational opportunity while also carrying athletic, personal, and later developmental meanings. For several HLSAs, these meanings shifted across their developmental histories as academic and athletic priorities changed and their student–athlete identities were repeatedly renegotiated.

### Instrumental rationality and educational access

Instrumental considerations were prominent in several participants' accounts of their early engagement with competitive sport. Athletic credentials were described as a practical means of expanding university admission opportunities within the highly competitive Gaokao system.

“*I practiced sports just to get into university; it was my only way out. Sports took me from a small county town to Peking University—a leap in social class that my family didn't even dare to imagine*.” (L05, Female)

Similar considerations appeared in other accounts. H02 recalled that her parents preferred the student-athlete pathway to entry into a professional provincial team because it offered greater educational security, while Y07 described obtaining the National First-Class Athlete certification as a central goal in her tennis participation. Athletic achievement carried clear practical value in educational decision-making, alongside athletic commitment, identity, family expectations, and meanings that changed over time.

### The dilemma of the “seesaw”: academic-athletic trade-offs

Participants repeatedly described academic study and competitive sport as difficult to sustain at the same level simultaneously. Their accounts emphasized shifting priorities, temporal conflict, and periods in which investment in one domain came at the expense of the other.

“*Growing up, it was always like a seesaw. If'academics' went up, ‘sports' would go down... Once I finally got good results in training, I would slack off in my studies. There were times it was incredibly difficult to maintain a balance between studying and training*.” (H02, Female)

Echoing H02's “seesaw” metaphor, F08 recalled that balance was virtually impossible during high school, leading him to “Completely throw away academics to focus entirely on practicing volleyball.” Such trade-offs were also physically demanding. X01 described “Running in minus 10 degrees in the dead of winter, striving for first place on the spot just to get into university, the challenge was simply too immense.” The demands involved substantial physical effort and difficult decisions about where to concentrate limited energy and attention.

### Negotiating the “in-between”: contextual identity tensions

The tension between academic and athletic demands was also reflected in how participants understood themselves across different settings. Some described feeling competent or visible in one domain while comparatively inadequate in the other.

“*As a student-athlete, I feel inferior in my studies, yet I don't stand out in training either. I feel my academics are worse than ordinary students, and my competitions are worse than professional athletes. I'm stuck in an awkward identity dilemma*.” (Y07, Female)

Similar contrasts appeared in other accounts. X01 described feeling “dazzling” on the sports field but “ordinary” in the classroom. H02 characterized her dual identity as “fragmented yet inseparable” and described having to “deliberately switch personalities and show ruthlessness” when entering the track environment. These narratives suggest that student and athlete identities could become more or less salient depending on the setting and the standards against which participants evaluated themselves.

Identity meanings were also capable of changing over time. Y15 described his trajectory as moving from a “utilitarian identity” toward “genuine love for the sport and recognition of the student-athlete value.” His account illustrates one possible form of identity reconstruction within the sample.

### Theme 2. Navigating the systemic labyrinth: uneven support, regulation, and performance-contingent recognition

Participants described substantial variation in the institutional conditions surrounding their academic and athletic development, including the availability of support, the organization of training and study, and the extent to which opportunities and recognition were tied to athletic performance.

### Conditional integration: support varies across institutional contexts

Participants described uneven coordination among families, coaches, schools, and other sources of support. In some accounts, these actors operated with different priorities, leaving athletes to manage academic and athletic demands with limited coordination.

“*My family wants to support me but doesn't understand training planning; my coach only cares about performance and ignores academics; and the school doesn't care about training at all*.” (H02, Female)

Differences in resource availability were also evident. L09 distinguished between “Category I” teams, such as basketball teams with corporate sponsors, and “Category II” teams, such as swimming, which she described as having “deficiencies in funding and guarantees.” Z11 similarly stated that “credits and opportunities are entirely bound to sports results; if we don't achieve rankings, we don't even get training credits.” Z10 described a comparable hierarchy within the team: “the strongest athletes enjoy all the resources—coaches' attention, others' respect, and exemption from chores, while the rest are ignored.”

Other accounts showed more supportive arrangements. Z11 described a school that provided a “specialized cafeteria” and arranged for “teachers to work overtime for remedial tutoring” when training conflicted with academic study. Z18 identified as a “beneficiary of ISE” and attributed this experience to access to “domestic top coaches, sports rehabilitation teams, and scientific guarantees ranging from technical analysis to nutritional supply.” Z11 likewise described the possibility of performing strongly in both domains:

“*I can complete an outstanding game on the court and write an excellent paper in front of the computer; this is the comprehensive ability brought by the integration of sports and education*.” (Z11, Female)

Such accounts contrasted with participants who continued to experience direct conflict between training and academic requirements. Y15 stated, “We are products of ISE, but training and studying are in perpetual conflict; true balance is incredibly difficult.” F08 also pointed to constraints at the school level, noting that “with only a dozen people playing volleyball in a school, it fundamentally cannot develop.” Z17 described how institutional priorities became especially visible when competitions and examinations overlapped:

“*The school says they support ISE, but as soon as a competition conflicts with an exam, they invariably prioritize sending us to the competition*.” (Z17, Male)

### Regulated lives and re-adaptation: adjustment continues across institutional transitions

Several participants described highly regulated training environments in which daily schedules, collective living, and training requirements structured much of their time. L09 recalled the “militaristic management” of her high school years, including a routine extending from “6:30 AM to 11:30 PM.” She described feeling “completely disciplined by the system” and pushed “near breakdown” because of the “extreme compression of energy” and the absence of meaningful rest time.

Participants did not interpret these environments in identical ways. Z04 described “closed training, unified schedules, and collective life” as conditions that required him to “become independent quickly and no longer rely on family.” At the same time, changes between institutional settings required further adjustment. Z11 observed that moving from a regular campus student-athlete setting into a university high-level sports team meant that “the management mode, training intensity, and interpersonal relationships have all changed, requiring a re-adaptation to the rules.” Structured training environments were experienced differently, while institutional transitions required renewed adjustment to routines, expectations, and relationships.

### Performance-contingent recognition: opportunity and status shift with athletic standing

A further recurring pattern concerned the relationship between athletic performance and access to opportunities, attention, and status within teams. L14 described both professional and university sports teams as operating according to a “law of the jungle,” where athletes with “poor performance or younger age are easily ignored and marginalized.”

F08 reported that because his “height was not an advantage” in the coach's evaluation, his “training opportunities and playing time were compressed.” C16 described both a decline in team status and a prolonged injury-related interruption to training. Elsewhere, C16 described an injury that interrupted training for approximately 1 year.

“*I dropped from being a starting player to the margins of the team, from the center to the periphery. I found that very difficult.”* (C16, Female)

“*At enrollment, I was soft-hearted and let an opponent win (conceded a point) finishing second in the group. Because of that, I lost my starting position on the school team, never had the chance to play again, and was completely marginalized by the team*.” (Y07, Female)

### Theme 3. Navigating uncertainty, informal practices, and multidimensional challenges

Their accounts differed in how they understood the relationship between talent and effort, responded to practices they regarded as unfair, and dealt with injuries, fear, and academic difficulties.

### Talent-effort tensions: perceived control over athletic outcomes varies

Several participants reflected on the extent to which athletic performance could be improved through effort and where they perceived limits beyond individual control. Their accounts did not converge on a single view of the relationship between talent and effort.

“*Some people are simply born to run; no matter how hard I train, I can't catch up to them. Talent is the ceiling, and effort can only get you closer to that ceiling*.” (H12, Female)

Similar concerns appeared in other accounts. Z17 noted that at the highest levels, “Everyone is a high-talent athlete; only then does effort become the deciding factor.” X01 described the psychological difficulty of “Not being able to directly convert hard work into athletic results.” C16 likewise identified “Injuries, draw results, and daily form” as factors affecting competitive outcomes and concluded that “the result is absolute, but effort does not guarantee a return.” Other participants interpreted the relationship differently. Z18 stated, “I don't believe in absolute talent gaps... diligence can compensate for clumsiness; effort itself is a kind of talent.”

### Unfairness beyond competition: informal practices and unequal resource access

Participants also described experiences in which formal expectations of competition and resource allocation did not correspond with what they encountered in practice. Some accounts concerned direct interference in competition.

“*When we played in the National High School League, the ‘experts' said the host team had to win the championship and demanded that we ‘act' (throw the game). In the final point, the expert ordered us to intentionally serve the ball directly out of bounds... we were much better than them, but we were forced to lose*.” (F08, Male)

F08's account illustrates an experience in which the expected fairness of competition was overridden by an externally imposed outcome. Similar concerns appeared in participants' accounts of routine resource allocation. This pattern resembles the “Matthew Effect” (Merton, 1968), whereby those who are already advantaged gain further access to resources and support. L05 shared that, “Coaches never give supplements to me; they only give them to athletes with good results… resources are completely skewed toward top players.” Z06 similarly described unequal coaching attention, noting that “The good get better and the bad get worse… there is no fairness at all.” Participants linked athletic achievement to expanded educational and, in some cases, occupational opportunities. Some participants thus linked stricter admission standards to fairness, qualification, and student-athlete legitimacy.

### Challenges cross domains, responses diverge

Participants described a range of difficult experiences arising from injury, continued participation under physical strain, fear following injury, and adjustment to academic demands.

“*I've had two surgeries on my leg. My family didn't want me to play anymore and wanted me to dedicate 70% to studying and 30% to training. But I constantly rebelled and insisted on playing*.” (Z10, Female)

Participants also described psychological consequences that persisted beyond the immediate physical event. L03 reported that, even after recovering from a broken nose, she continued to experience “fear of the ball hitting my face again,” which affected her subsequent performance. Academic transitions created a different form of difficulty. Z04 recalled struggling in university English classes and described the experience of “understanding absolutely nothing” as a “complete collapse.” Some participants retrospectively connected sport with persistence, self-understanding, direction, and perceived competence in later challenges.

### Theme 4. Beyond the field: perceived educational and occupational mobility, identity legitimacy, and developmental meanings

Participants' retrospective accounts of opportunities associated with athletic achievement, responses to social perceptions of student-athletes, and the longer-term meanings they attributed to sport. Some participants described sport as widening educational or occupational opportunities, while others emphasized changes in self-understanding, coping, and perceived life competence.

### Perceived educational and occupational mobility: the conversion of sports capital

Several participants described athletic achievement as widening their educational and, in some cases, subsequent professional opportunities. Their accounts concerned access to selective universities, postgraduate study, and later employment.

“*I used my national record in the 400m hurdles to secure admission to Peking University, converting my athletic capital into academic credentials and achieving a jump from an ordinary high school to a top-tier university*.” (X01, Male)

L03 similarly described football as her “core capital for graduate admission and eventually securing a university faculty position.” Z04 reflected on the importance of sport within the highly competitive examination environment in Shandong, describing athletic achievement as having “compensated for insufficient academic capital” and helped him enter a “985” university. Participants understood their athletic achievements as having expanded educational and, for some, later occupational opportunities within their own life trajectories.

### The “shortcut” stigma and legitimacy claims: contesting student-athlete stereotypes

Participants also described encountering social perceptions that questioned the legitimacy or academic competence of student-athletes. One recurring example was the view that admission through athletic achievement represented an easier “shortcut” into university.

“*Teachers think regular students have it harder in Gaokao... they don't understand the physical and psychological damage we endure. In competitive sports, there is no such thing as ‘health'*.” (L03, Female)

Y07 similarly referred to a “simplified social image” of student-athletes. L05 recalled that classmates avoided working with her in group projects because of the perception that sports students were “unorganized and drag the team down.” Participants responded to such perceptions in different ways, including directly challenging stereotypes and emphasizing the demands associated with competitive sport.

Some participants also discussed recent admission reforms when reflecting on the legitimacy of the high-level sports team pathway. L05 described stricter admission requirements as changing the pathway from a “policy dividend” into an “ability screening,” while Y15 argued that the reforms could help ensure that those entering the pathway possessed “genuine professional quality.” These accounts show how some participants interpreted policy changes in relation to questions of fairness, qualification, and the legitimacy of their student-athlete status.

### Sport acquires meaning beyond performance

Beyond educational access and competitive achievement, some participants retrospectively attributed broader personal meaning to their sporting experiences.

“*The 400m hurdles is inherently challenging; everyone is exhausted by the final stretch, but you have to finish. This process tempers my mind... it allows me to face social or life challenges with that same spirit—never giving up and fighting to the end*.” (X01, Male)

H02 similarly described being “psychologically tempered” through training, injury, and exhaustion and stated that sport taught her to be “mature enough not to cry when facing difficulties.” Z06 described the student-athlete experience as contributing to his sense of becoming a “hexagon warrior” with “unlimited potential.” L09 used a different metaphor, describing swimming as a “lawnmower” that had cleared a path through life and helped her avoid “taking unnecessary detours.” These accounts indicate that some participants retrospectively connected sport with persistence, self-understanding, direction, and perceived competence in dealing with later challenges.

## Discussion

DC development provides the broader developmental frame for this discussion, while conditional inclusion serves as the integrative concept connecting the empirical patterns identified across the four themes. Role identity, attribution and appraisal, and Self-Determination Theory illuminate specific psychological processes within these patterns. Bourdieu's concept of capital conversion and the Matthew Effect are used more narrowly to interpret educational access and cumulative differences in recognition and resources.

### From role balance to identity work: negotiating competence and legitimacy across contexts

Dual-career research often treats academic and athletic roles as concurrent commitments that athletes must coordinate (Stambulova et al., 2015; Ryba et al., 2017). H02's “seesaw” captured fluctuating investment between study and sport, but Y07's account went further: she evaluated herself as academically weaker than ordinary students while also falling short of the athletic standard represented by professional athletes. X01 likewise described feeling “dazzling” on the sports field but “ordinary” in the classroom. The difficulty lay partly in locating competence across settings governed by different standards of success.

This contextual movement is important because role identity is tied to how individuals interpret their competence and future possibilities. In a recent Frontiers in Psychology study of intercollegiate student-athletes, Chen and Wang (2026) found that stronger student identity was associated with higher general self-efficacy and a longer planning horizon, while reflected appraisal strengthened the relationship between student identity and self-efficacy.

Their findings link role identity to self-evaluation and perceived capability. H02's description of being “fragmented yet inseparable” and deliberately altering her demeanor on entering the track suggests that competence and role expression were recalibrated across academic and athletic settings. Identity negotiation involved interpreting what each context implied about one's competence.

External evaluation further complicated this process. Chen and Ni (2024), also writing in Frontiers in Psychology, showed that sport-related identities among Chinese students could be valued in sporting contexts yet devalued in academic settings, with some participants concealing their sport-related identity to avoid negative categorization. Their analysis highlights the context dependence of stigma and the psychological importance of how others classify sport-related identities. The HLSAs in the present study encountered a related but more specific challenge to legitimacy. The “shortcut” stereotype reported by L03 and assumptions about academic inadequacy described by L05 did not simply question academic ability; they questioned whether athletic achievement constituted a legitimate basis for university membership. Participants' responses included direct rebuttal, reference to the demands of competitive sport, and, in some cases, interpretation of stricter admission standards as evidence that the pathway required genuine athletic and academic qualification. These responses are better understood as identity work around legitimacy.

The Chinese admissions context makes this legitimacy issue particularly salient. Athletic achievement could function simultaneously as a basis of athletic identity and as a resource for educational access. Bourdieu's (1986) concept of capital conversion helps explain how athletic competence can facilitate educational access. Once athletic competence contributes to entry into selective higher education, judgments about the value of that competence can extend into judgments about whether the athlete belongs there. This also helps explain why an initially instrumental relationship with sport need not remain psychologically fixed. Y15's retrospective shift from a “utilitarian identity” toward genuine attachment to sport illustrates how the meaning of the athlete role could be revised as its connection to competence, belonging, and personal value changed over time.

### When effort does not guarantee outcomes: attribution, appraisal, and coping

A particularly salient psychological tension concerned the limits of personal control over athletic outcomes. Participants did not simply distinguish between “talented” and “hard-working” athletes. Instead, they differed in how they understood the causal relationship between talent, effort, and performance. H12 described talent as a ceiling that effort could approach but not breach; X01 emphasized the psychological difficulty of sustaining hard work when it does not produce corresponding improvements in results; and C16 identified injury, competition draws, and daily form as sources of uncertainty that cannot be controlled. Z18 took a different position, rejecting the idea of an absolute talent gap and redefining sustained effort itself as a form of talent. The disagreement centered on how much control athletes believed they retained when effort no longer reliably predicts outcomes.

Weiner's attribution framework distinguishes explanations of success and failure along three dimensions: locus, stability, and controllability (Weiner, 1985). Ability is commonly construed as relatively stable and less controllable, while effort is categorized as more controllable. The participants' accounts did not map neatly onto this established distinction. At high levels of competition, effort remains personally controllable, but its outcomes do not: physical limits, injury, competitive circumstances, and day-to-day condition can all break the expected link between effort investment and performance outcome. These attributions shape expectations, emotional responses, and persistence. Attributional processes in athletes are associated with fear of failure, self-criticism, and self-efficacy under performance pressure (Huang et al., 2025).

Z18 reframed effort itself as part of ability, while other participants adapted to the reality of limited control by accepting that effort can increase the probability of strong performance without guaranteeing a specific result. These accounts suggest that psychological adaptation to uncertain outcomes often involves revising causal expectations, and adaptation involved revising causal expectations when experience disrupted a simple effort-to-outcome relationship.

The transactional model of stress and coping emphasizes appraisal of demands and coping resources (Lazarus and Folkman, 1984). Sport research has similarly shown that perceived control and challenge-to-threat appraisal shape how athletes respond to competitive stress (Gomes et al., 2022), while qualitative studies of injured athletes have identified attribution, cognitive restructuring, social support, withdrawal, and other coping responses during recovery (Leguizamo et al., 2023). Z10 continued investing in sport after two surgeries despite family pressure to reduce training; L03 described persistent fear of re-injury after her broken nose had fully healed physically; and Z04 characterized his inability to follow university English classes as a “complete collapse.” Participants showed heterogeneous processes of attribution, perceived control, appraisal, and behavioral or emotional response. The psychological challenge of DC lies partly in continuing to act when effort remains necessary but no longer guarantees outcomes.

### Conditional adaptation: psychological needs within uneven institutional environments

Participants' accounts differed markedly in the level of support available alongside their training and study commitments. Some described fragmented expectations across family, coaches, and school, whereas others reported access to remedial teaching, specialized nutrition support, sports rehabilitation, and coordinated academic assistance. Li et al. (2026), who studied 339 elite track-and-field athletes, differentiated four distinct types of support: emotional, esteem, informational, and tangible. Their analysis found that esteem and tangible support showed the strongest associations with athletic engagement. Role identity also partially mediated these associations, linking support to perceived role value and group identification. Participants' accounts included tangible and informational support such as tutoring, rehabilitation, nutrition, and technical assistance, while uneven coaching attention reflected differences in esteem-related support.

Self-Determination Theory (SDT) distinguishes autonomy-supportive from controlling environments. Participant L09 described a tightly regulated training schedule with almost no discretionary time or rest, whereas Participant Z04 associated “closed training, unified schedules, and collective life” with becoming more independent. This contrast suggests that regulation carries psychological impact because of the experiences athletes have within that structured context. Chen et al. (2026) studied 800 Chinese university student-athletes and found that autonomy-supportive coaching correlated with lower athlete burnout, while controlling coaching correlated with higher burnout; autonomy, competence, and relatedness partially mediated both associations. Of the three basic psychological needs, competence showed the strongest indirect effect. Highly structured training environments can differ dramatically in whether athletes experience meaningful choice, consistent recognition of their competence, and supportive interpersonal relations. The relevant distinction for DC adaptation is the psychological quality of the environment in which regulation occurs.

The most distinct tension identified in the present data concerns whether competence is socially recognized. When athletes sustain injuries, are perceived as physically disadvantaged, or no longer perform at the expected level, their playing time, coaching attention, allocated resources and team status may all change. Self-Determination Theory (SDT) identifies competence, relatedness, and autonomy as three basic psychological needs: competence refers to the experience of being effective in activities one values, while relatedness refers to the experience of connection and belonging in important interpersonal relationships (Deci and Ryan, 2000). The concentration of recognition and resources among athletes who already held stronger team positions also resembles the cumulative advantage described by Merton's (1968, 1988) Matthew Effect. For athletes whose team standing declines, reduced opportunities thus correspond to both fewer resources and weaker social recognition of their competence. Formal participation in both domains did not guarantee stable experiences of autonomy, competence, or relatedness. HLSAs could remain enrolled while reporting restricted discretion, unstable recognition, or reduced belonging when opportunities were closely tied to current athletic standing. These accounts position DC adaptation within the institutional conditions through which support, recognition, and discretion are experienced across academic and athletic roles.

We conceptualize conditional inclusion as a form of dual-career participation in which formal access to higher education is secured while legitimacy, recognition, support, and developmental opportunity remain contingent on changing academic and athletic evaluations, particularly current athletic standing. Performance-contingent recognition is one mechanism through which this conditionality operates; institutional support refers to the resources and opportunities available within these conditions; and DC adjustment refers to how athletes interpret and respond to them.

## Limitation

The study focused on HLSAs who had successfully entered higher education through the high-level sports team pathway. Retrospective accounts may also be influenced by recall and voluntary self-selection. Participants entered university across a period of substantial policy change, which broadened the temporal range of the accounts. Institutional conditions were examined as experienced and interpreted by HLSAs rather than as independently verified organizational practices. The first author's insider position formed part of the interpretive process. Because family income, parental education, and other socioeconomic indicators were not collected, mobility refers to participants' perceived educational and occupational opportunities. Full interview transcripts cannot be made publicly available because of re-identification risks.

## Conclusion and implications

Athletic achievement can open access to higher education without securing stable legitimacy or belonging within it. Participants continued to negotiate competence and legitimacy across academic and athletic settings, while stereotypes and performance-based judgments shaped how their student-athlete status was recognized. This instability also appeared in how athletes understood control over performance. Effort often remained within their control even when its return did not, as injury, bodily limits, competitive conditions, and day-to-day form disrupted the expected relationship between effort and outcome. Adaptation involved recalibrating causal expectations and deciding what could still be changed.

Recognition and support were similarly experienced as unstable when athletic standing changed. Playing opportunities, coaching attention, resources, and team status could fluctuate, making institutional arrangements part of participants' experiences of competence and belonging. Across these accounts, access, control, recognition, and developmental opportunity did not necessarily stabilize together.

These intersecting patterns are captured by the concept of conditional inclusion. For ISE, the practical issue is whether opportunities opened through sport can extend beyond continued dependence on athletic performance. Academic structures that broaden competence, identity, and future options beyond sport offer one institutional route for doing so.

Concentrating HLSAs within narrowly sport-specific academic tracks may reinforce their separation from the broader university environment. Universities could instead develop interdisciplinary structures that expand academic exposure while accommodating intensive athletic commitments. At Tsinghua University, HLSAs admitted through the high-level sports team pathway enroll in the “Physical Education+Business Administration” dual bachelor's degree program, which integrates physical education with business and management training (Tsinghua University, 2026). Peking University has adopted a different interdisciplinary model through its Physical Education (Journalism and Communication) program, jointly developed by the School of Journalism and Communication and the Department of Physical Education. HLSAs admitted through the high-level sports team pathway follow a 5-year study arrangement combining physical education, sports training, and journalism and communication studies (Peking University, 2024, 2026). Both models illustrate how interdisciplinary curriculum design can widen HLSAs' academic exposure beyond narrowly sport-specific pathways.

In this sense, the “systemic labyrinth” faced by Chinese HLSAs is formed not only by the intersecting demands of admission, training, study, resource allocation, recognition, and opportunity, but also by how these conditions are psychologically experienced through questions of competence, legitimacy, control, and belonging. The “dual-track breakthrough” is therefore more than the achievement of balance between sport and education. It lies in turning athletic achievement into broader academic and career possibilities while also reconstructing the meaning of sport, self, and future beyond immediate performance. For ISE, the deeper task is to create conditions in which sporting achievement opens the university door but does not define the limits of what student-athletes can subsequently become.

## Data Availability

The datasets presented in this article are not readily available because the interview transcripts are not publicly available because they contain detailed contextual information that could compromise participant anonymity. De-identified analytic materials may be made available upon reasonable request, subject to applicable ethical and confidentiality requirements. Requests to access the datasets should be directed to 2020210083@bsu.edu.cn.
