# Supplementary material for: Navigating the systemic labyrinth: the dual-track breakthrough of student-athletes under China's “Integration of Sports and Education”
Source: Front Psychol. 2026 Sep 29;17:1882691. doi: 10.3389/fpsyg.2026.1882691 (PMC13657933; doi:10.3389/fpsyg.2026.1882691)
Supplement: Supplementary file 1 [file Supplementary_file_1.docx]

**Supplementary Appendix A. Final Semi-Structured Interview Guide**

**Development of the interview guide**

The semi-structured interview guide was developed iteratively. The initial version was organized around three broad domains—participant background, developmental experiences, and meaning/value—with attention to key people, events, and settings within participants’ developmental histories. Following discussions with experts, teachers, and peers, the second version strengthened its developmental-history orientation by adding prompts concerning influential people, turning points, and significant settings. Two pilot interviews indicated a need to better capture recurring experiences across participants, non-linear trajectories, and participants’ own understandings of developmental stages. The final guide therefore retained the original domains while adding questions concerning group characteristics, policy and institutional contexts, and retrospective stage construction.

Two pilot interviews were then conducted. The results of these interviews indicated that the guide needed greater sensitivity to crecurring experiences across participants, non-linear developmental trajectories, and participants’ own understandings of their developmental stages. The final version therefore retained the three original domains, while adding questions covering group characteristics, policy and institutional contexts, and retrospective stage construction. Core questions structured each interview, and follow-up prompts were used to encourage participants to elaborate on relevant experiences.

| ****Dear participant,**** This interview aims to understand the experiences and developmental trajectories of high-level student-athletes. Your responses will be used anonymously for research purposes. Your identity will be kept confidential, and the information you provide will be handled in accordance with the study’s ethical and confidentiality procedures. Please answer according to your own experiences and views. | |
| --- | --- |
| ****Background information collected****  **Undergraduate institution; undergraduate major; highest educational qualification; institution type; sport specialization; current occupation/status; and highest competitive achievement.** | |
| **Core question** | **Planned follow-up prompts** |
| **1. What role did your family play in your decision to pursue sport?** | Did any family member particularly influence your choice of sport? Was there any experience that changed your decision? |
| **2. How did you balance academic study and training? What support or difficulties did you experience from your school and teachers?** | Were there any particular forms of support or difficulty in classes, examinations, or academic arrangements? |
| **3. How did the sporting environments in which you participated—such as school teams, clubs, sport schools, or local teams—support or constrain your athletic development?** | Were there particular rules, resources, or resource-allocation practices that left a strong impression on you? |
| **4. What sporting experiences or events have been particularly memorable in your development?** | Did a particular coach or teammate strongly influence you? Did a competition, injury, selection, or non-selection become a turning point? |
| **5. If you were to divide your sporting career into several stages, how would you divide it?** | What characterized each stage? What was the greatest challenge during each stage? |
| **6. Looking back, what was the biggest change you experienced around the time you entered university?** | How did you experience and interpret that change? |
| **7. Have you experienced situations in which you felt unfairly treated or misunderstood? What impact did these experiences have on you?** | Did anyone support you or disappoint you in such situations? |
| **8. How do you understand your dual identity as both a student and an athlete?** | What kinds of pressure or uncertainty, if any, have these two roles created? Where did these pressures mainly come from? |
| **9. What does sport mean within your broader life plans?** | In what ways has being an athlete shaped your understanding of your own value or future? |
| **10. What characteristics do you think distinguish high-level student-athletes as a group?** | In what ways do you think you differ from students who are not high-level athletes? |
| **11. What do you think is the greatest misunderstanding outsiders have about high-level student-athletes?** | How would you prefer others to understand this group? |
| **12. In your previous experiences, have you perceived the presence of the Integration of Sports and Education (ISE)? Please give examples.** | What would genuine integration of sport and education look like from your perspective? |
| **13. What do you think has changed most following reforms to the high-level sports team admission system?** | What effects, if any, do you think these reforms have had on athletes, peers, or universities? |
| **14. If you could choose, would you prefer to major in Physical Education or in another academic field? Why?** | Do you think your university provides academic and developmental support appropriate to the needs of student-athletes? |
| **15. If you had to use three keywords to describe your developmental trajectory, which would you choose?** | Do you think these keywords also reflect characteristics shared by other high-level student-athletes? Why or why not? |
| **16. What advice would you give to students who hope to become high-level student-athletes in the future?** | Are there any particularly important experiences or lessons that you would want to pass on? |
| **17. Is there anything important about your experience that I have not asked about but that you would like to add?** | Open follow-up as appropriate. |

**Supplementary Appendix B. Illustrative Analytic Process and Reflexive Theme Development**

**Supplementary Table B1. Application of the six-phase RTA process in the present study**

| **RTA phase** | **Analytical actions in the present study** | **Main analytical outcome** |
| --- | --- | --- |
| **1. Familiarization with the data** | The first author repeatedly read the verbatim Chinese transcripts while returning to the original audio recordings where necessary. Initial observations were recorded using the Comment function in Microsoft Word, including notes on recurring experiences, contradictions, contextual details, and relevant paralinguistic features such as pauses, silences, and changes in tone. At this stage, the purpose was to become closely familiar with each participant’s account rather than to apply a predetermined theoretical framework. | Preliminary observations and case-level reflexive notes |
| **2. Generating initial codes** | The transcripts were transferred into a structured Microsoft Excel workspace containing parallel fields for data extracts, initial codes, and analytical/reflexive memos. Initial coding was conducted by the first author and was predominantly **inductive and semantic**, remaining close to what participants explicitly described. Codes were revised, split, combined, or relabeled as the researcher moved repeatedly between individual extracts and the wider dataset. Theoretical concepts were not used as a priori coding categories. | Data-near initial codes and early analytical memos |
| **3. Generating candidate patterns and sub-themes** | Initial codes were compared within and across cases using Excel sorting, filtering, and manual grouping. Related codes were provisionally clustered according to shared patterns of experience, meaning, action, and context. At this stage, analysis began to move beyond explicit description toward relationships among experiences and their possible underlying meanings. Through repeated comparison and regrouping, 14 provisional sub-themes were developed. | Fourteen provisional sub-themes |
| **4. Reviewing and developing themes** | The 14 sub-themes were reviewed against both their supporting extracts and the full dataset. The first author returned to transcripts when candidate interpretations appeared too broad, overly negative, theoretically loaded, or insufficiently supported. Analytical memos were used to question early interpretations and to record decisions about retaining, narrowing, merging, or reframing candidate themes. Theme development became increasingly abductive and latent, with relevant theoretical concepts brought iteratively into dialogue with developing patterns as interpretive resources rather than used as a priori coding categories. | Refined sub-themes with clearer evidential and conceptual boundaries |
| **5. Defining, naming, and integrating themes** | The first author examined the relationships among the refined sub-themes and organized them into four overarching themes. Theme names and organizing concepts were revised to capture the central pattern represented by each theme without implying that all participants experienced an identical sequence. The 14 sub-themes were further reviewed for conceptual boundaries, wording, and evidential fit. Critical peer dialogue with the second author contributed to the refinement of seven sub-theme labels where the working formulations were overly absolute, conceptually conflated, or more theoretically expansive than the data supported. The first author subsequently examined relationships among the refined sub-themes and integrated them into four overarching themes. The four-theme structure was treated as an interpretive analytical framework rather than an empirically validated developmental-stage model. | Four overarching themes integrating the 14 sub-themes |
| **6. Producing the report** | Representative extracts were selected to demonstrate both recurrent patterns and meaningful variation across cases. Analysis and writing remained iterative, with claims repeatedly checked against the original Chinese transcripts. Quotations selected for publication were initially translated into English by the first author and subsequently reviewed and verified by the second author. Translation prioritized close correspondence with the original wording; minor adjustments were made where necessary for English readability without introducing analytical terminology into participants’ direct quotations. | Final analytical narrative supported by translated illustrative extracts |

**B2. Illustrative analytic trail**

Table B2 provides four illustrative analytic trails showing how selected data extracts were developed from data-near codes into broader interpretive patterns and, subsequently, into the final thematic structure. These examples are illustrative rather than exhaustive and are not intended to imply a fixed or mechanically reproducible coding pathway. Consistent with RTA, theme development involved iterative movement between individual extracts, the wider dataset, analytical memos, and developing interpretations.

**Supplementary Table B2. Illustrative progression from data extracts to final themes**

| Participant | Illustrative data extract | Data-near initial code(s) | Working sub-theme | Final overarching theme | Analytic development |
| --- | --- | --- | --- | --- | --- |
| **L05** | “At that time, I just wanted to get into university. There was nothing else to it. I wanted to get into university through sport.” | **Interest**  **→university-admission tool**  **→ skill** | **T10. Instrumental sport choices under instrumental rationality** | **Theme 1: The Paradox of Sports Capital: Instrumental Rationality and Dual-Track Identity Negotiation** | Comparison with other admission narratives shifted the interpretation from an individual educational choice toward a recurring pattern in which athletic achievement functioned as a resource for educational access. Capital conversion was subsequently brought into dialogue with this pattern as an interpretive lens. |
| **Z04** | “The environment was very closed off. When you went outside and talked with others, including about recent issues or current topics, you felt somewhat disconnected from society.” | **Closed environment and social disconnection** | **T11. Effects of the total institution and resocialization adaptation** | **Theme 2: Navigating the Systemic Labyrinth: Institutional Rigidity and Marginalized Survival** | Related accounts of spatial, temporal, and informational restriction broadened the initial focus on social disconnection. Goffman’s concept of the total institution was subsequently used to interpret these patterns rather than as an initial coding category. |
| **F08** | “Sport is not something where, if I practice twice as much as someone else, I will necessarily become better than them... some people understand it after two or three attempts, while I might not get there after one or two years.” | **Perceived futility of effort** | **T14. Binary perceptions of the absoluteness of talent and relativity of effort** | **Theme 3: The Hidden Curriculum of the Arena: Cognitive Binaries, Moral Gray Areas, and Pragmatic Adaptation** | Cross-case comparison revealed different interpretations of the limits of effort, ranging from resignation to active reframing. The analysis therefore developed into a tension between perceived innate limits and the relative value of effort rather than a single fatalistic position. |
| **L03** | “Some teachers think ordinary students have a harder Gaokao and that we took a shortcut... I argued with them: do you know how much physical and psychological hardship we went through in training before we could enter a university like this?” | **Rebutting the “shortcut” stereotype / external prejudice** | **T9. Elite contestation and social image** | **Theme 4: Beyond the Field: Elitist Contestation, Policy Agency, and Holistic Human Development** | Similar accounts of academic inferiority stereotypes and the perception of athletic admission as a “shortcut” were compared across cases. Participants’ responses ranged from disengagement to active rebuttal, supporting an interpretation centered on stigma contestation and identity legitimacy. |

**B3. Reflexive Refinement of Theme Interpretations**

Reflexive refinement occurred throughout theme development. The first author used analytical memos to question emerging interpretations, while later critical peer dialogue with the second author was used to challenge conceptual boundaries, wording, and evidential fit. These processes prompted reconsideration of interpretations that were overly absolute, theoretically expansive, conceptually conflated, or insufficiently sensitive to variation across participants.

One example concerned L05. An early analytical memo interpreted L05’s declining athletic status, weakened academic identification, and perceived stigma through the concepts of learned helplessness and “hollow-heart” distress. On returning to the transcript and comparing this interpretation with other cases, the first author judged these labels to be more clinically and theoretically loaded than the evidence warranted. They were therefore not retained, and the interpretation was narrowed toward more directly supported patterns of performance-contingent marginalization, identity tension, instrumental motivation, and stigma.

**Supplementary Table B3. Illustrative examples of reflexive refinement during theme development**

| **Working sub-theme** | **Earlier formulation** | **Reflexive concern** | **Revised formulation** | **Analytical consequence** |
| --- | --- | --- | --- | --- |
| **T1** | Growth trajectories and stage characteristics driven by dual-track negotiation | The wording implied that dual-track negotiation uniformly drove all developmental choices and stages. | Growth trajectories and stage characteristics primarily shaped by dual-track negotiation | The revision retained dual-track tension as a major organizing pattern while avoiding a single-cause explanation of participants’ developmental histories. |
| **T2** | Environmental ecology and multiple resource-allocation mechanisms | “Environment” and “resource allocation” were treated as parallel concepts despite operating at different analytical levels. | Ecological environment under multiple resource allocations | The interpretation was reorganized so that resource allocation became a mechanism within the broader developmental ecology rather than a separate equivalent construct. |
| **T3** | Dynamic changes in identity and psychological landscape | Identity and “psychological landscape” were conceptually broad and not positioned at the same analytical level. | Dynamic psychological changes centered on identity | The revised interpretation placed changes in self-definition and identity negotiation at the center while treating other psychological experiences as related rather than co-equal constructs. |
| **T6** | Sport policy literacy and anticipated group-level effects | “Policy literacy” introduced a normative and standardized concept that exceeded participants’ own accounts, which mainly concerned perceptions, evaluations, and responses to policy change. | Group perceptions of and responses to sport policy | The revised formulation moved closer to the data by emphasizing policy recognition, critical interpretation, and subsequent action rather than attributing a predefined form of “literacy.” |
| **T9** | Social construction of group characteristics and elite contestation | “Social construction” was considered too broad a sociological abstraction for data primarily concerning social image, perceived stigma, and participants’ responses. | Elite contestation and social image | The revision narrowed the interpretation to how HLSAs perceived, contested, and attempted to reshape public representations of their group. |
| **T11** | Cognitive closure and resocialization pain under a total institution | The wording privileged a uniformly negative interpretation and reduced the effects of institutional enclosure to cognitive restriction and distress. | Effects of the total institution and resocialization adaptation | The revised formulation allowed both constraining effects and adaptive responses to be represented, reducing negative narrative bias. |
| **T12** | Rigid management and marginalization within high-level sports teams | “Marginalization” could be read as referring to the team as an institution rather than to individual athletes, creating ambiguity in the object of analysis. | Rigid management of high-level sports teams and individual marginalization | The revision clarified the proposed relationship between rigid internal management, gaps in athlete development, and experiences of individual marginalization. |

Other working sub-themes were likewise revised as additional cases were incorporated. For example, an early interpretation of the talent–effort relationship as “effort futility and fatalism” was later broadened to capture a more differentiated tension between perceived innate limits and the relative value of effort, while interpretations of informal practices in sport were expanded to include variation in fairness experiences.
